# A Situational Analysis of the Impact of the COVID-19 Pandemic on Digital Health Research Initiatives in South Asia

**DOI:** 10.7759/cureus.48977

**Published:** 2023-11-17

**Authors:** Akash Prabhune, Sachin Bhat, Aishwarya Mallavaram, Ayesha Mehar Shagufta, Surya Srinivasan

**Affiliations:** 1 Health and Information Technology, Institute of Health Management Research, Bangalore, IND; 2 Health, Public Affairs Centre, Bangalore, IND

**Keywords:** telehealth, india, mobile apps (mhealth), data science, surveillance, covid-19, sustainability assessment, digital health

## Abstract

The objective of this paper was to evaluate and compare the quantity and sustainability of digital health initiatives in the South Asia region before and during the COVID-19 pandemic.

The study used a two-step methodology of (a) descriptive analysis of digital health research articles published from 2016 to 2021 from South Asia in terms of stratification of research articles based on diseases and conditions they were developed, geography, and tasks wherein the initiative was applied and (b) a simple and replicable tool developed by authors to assess the sustainability of digital health initiatives using experimental or observational study designs.

The results of the descriptive analysis highlight the following: (a) there was a 40% increase in the number of studies reported in 2020 when compared to 2019; (b) the three most common areas wherein substantive digital health research has been focused are health systems strengthening, ophthalmic disorders, and COVID-19; and (c) remote consultation, health information delivery, and clinical decision support systems are the top three commonly developed tools.

We developed and estimated the inter-rater operability of the sustainability assessment tool ascertained with a Kappa value of 0.806 (±0.088).

We conclude that the COVID-19 pandemic has had a positive impact on digital health research with an improvement in the number of digital health initiatives and an improvement in the sustainability score of studies published during the COVID-19 pandemic.

## Introduction and background

The Global Digital Health Strategy 2020-2025 of the World Health Organization (WHO) defines digital health as the field of knowledge and practice associated with the development and use of digital technologies to improve health [1]. The fit between individuals, task, and technology (FITT) framework developed by Ammenwerth et al. [2] for the classification of information technology initiatives broadly classified digital health initiatives into three tasks: health information exchange (HIE), data science, and surveillance [3]. The WHO’s Global Digital Strategy stated its vision as “improving health for everyone, everywhere by accelerating the development and adoption of appropriate, accessible, affordable, scalable, and sustainable person-centric digital health solutions.” The vision document has highlighted four strategic objectives: (a) institutionalization of digital health in the national health system, (b) integration of strategy to ensure the success of digital initiatives, (c) promotion of appropriate use of digital technologies for health, and (d) addressing the impediments faced by least developed countries implementing digital health technologies.

The COVID-19 pandemic disrupted the global healthcare systems, the increasing COVID-19 cases diverted the available healthcare infrastructure, resources, and manpower toward controlling the pandemic, and the delivery of normal healthcare services related to maternal care and childcare and non-communicable diseases was disrupted [4-7]. The disruption in the normal delivery of healthcare services presented an opportunity for digital health initiatives to fill in the gap, with as many as 264 research papers on digital health indexed in PubMed in the year 2020 from the South Asia region as compared to 82 research papers indexed in the year 2019. The threefold increase in Medline-indexed research papers implies an increased interest in medical practitioners, researchers, and academicians in digital health from developing countries such as India, Bangladesh, Vietnam, Thailand, Sri Lanka, and Indonesia.

An increase in quantity is one aspect of the global digital health vision; the quality of research undertaken and key steps taken in the development phase to ensure the sustainability of the initiative are the two aspects that are explored to a limited extent.

The proposed situational analysis covers the abovementioned aspects of digital health research in South Asian countries, which are home to about a quarter of the total world population. This research paper aims to systematically analyze the change in patterns measured through a review of published literature before and during the COVID-19 pandemic.

This article was previously posted to the Research Square preprint server on June 7, 2021.

Objective

The objective of this paper was to evaluate and compare the quantity and sustainability of digital health initiatives in the South Asia region before and during the COVID-19 pandemic.

Methods

A comprehensive search strategy was developed using search terms digital health, mHealth, telehealth, e-health, health information exchange, data science, and surveillance with appropriate bullion operators “AND/OR.” The search was limited to the Medline database and restricted to South Asia, the English language, and within five years (2016-2020).

The search terms were as follows: (((((Digital Health) OR (mHealth)) OR (telehealth)) OR (e-health)) AND (((Health information exchange) OR (Data Science)) OR (Surveillance)) AND (y_5[Filter])) AND (((South East Asia) OR (India)) OR (South Asia) AND (y_5[Filter])).

The study has been divided into two sections. The first section is (a) a descriptive analysis of the number of research articles published on digital health. All the search results will be screened for eligibility based on any research article published from January 2016 to March 2021, and the research articles must be focused on either health information exchange, data science, or surveillance tasks using digital tools. The descriptive analysis will include the number of research articles published year on year from January 2016 to March 2021, the stratification of research articles based on diseases and conditions they were developed, geography, and tasks wherein the initiative was applied.

The second section is the assessment of sustainability. We used the sustainability definition given by Hallin et al. [8] as “the ability to generate or gain access to the resources, financial or otherwise, needed to protect and increase the value of the content or service for those who use it.” The eligibility criteria for the assessment of quality are as follows: research articles published between January 2016 and March 2021, research articles wherein the focus was on the development, validation, and scaling up of the digital initiative, and research articles using randomized controlled trials and quasi-experimental, case-control, cross-sectional, diagnostic, and pilot study designs. The exclusion criteria for the assessment of quality are research articles including review articles, policy articles, letters to editors, and correspondence and economic evaluation studies and qualitative analysis studies.

**Figure 1:**
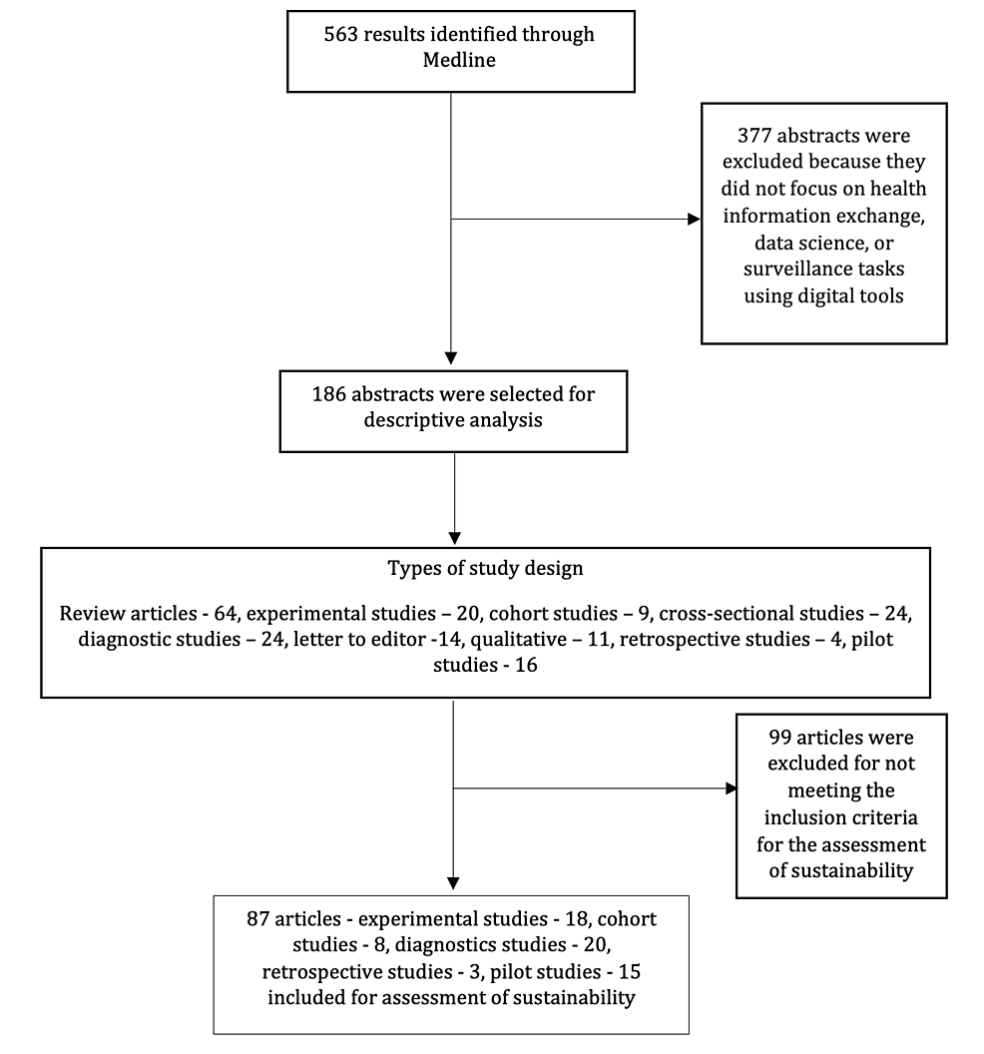
PRISMA flow diagram for the selection of articles for the study PRISMA: Preferred Reporting Items for Systematic Reviews and Meta-Analyses

A review of various tools available for the assessment of sustainability across various sectors such as manufacturing, corporate management, multisectoral sustainability assessment tool, program sustainability assessment tool, and clinical sustainability assessment tool developed by the Center for Public Health Systems Sciences, Washington University, St. Louis, was conducted. However, none were found suitable to assess the sustainability of digital health initiatives; thus, a simple tool was developed based on the objectives and vision stated in the Global Digital Health Strategy 2020-2025 and the principles of Public Health Data Standards [8-14]. Two independent raters (AM and SB) reviewed the eligible studies for three key areas: (a) multisectoral and cross-sectoral engagement, (b) standards and interoperability, and (c) people-centric approach. The sustainability score for the study was determined as the average score given by two independent raters. Table 1 presents the detailed tool used for assessing sustainability, and Figure 1 presents the detailed framework for the selection of studies.

**Table 1:** Tool of assessment of sustainability ASTM: American Society for Testing and Materials, HIPAA: Health Insurance Portability and Accountability Act, HL7: Health Level 7, ISO: International Organization for Standardization, LOINC: Logical Observation Identifiers Names and Codes, SNOMED: Systematized Nomenclature of Medicine, UMLS: Unified Medical Language System

Study citation			
Section A: Multisectoral and cross-sectoral engagement	Q1: Does the study involve a multisectoral team?	Consider: (a) Were all the authors from the medical field only, and (b) are the data scientists/development engineers/IT consultants acknowledged in the paper?	Yes, no, can’t say (if your answer is yes, +; no, -1; and can’t say, 0)
	Q2: Does the study involve engagement with sectors other than health?	Consider: (a) With respect to the study objective, did the study mention any other sectors such as nutrition or agriculture, and (b) were the study endpoints or outcome assessment involved data from other sectors?	Yes, no, can’t say (if your answer is yes, +; no, -1; and can’t say, 0)
Section B: Standards and interoperability	Q3: Does the study mention adherence to any standards of data components, data interchange, and application-level support?	Consider: (a) Data components - reference information model, data elements, data types, and terminology (standards: HL7, SNOMED, LOINC, and UMLS), (b) data interchange - structured and free form documents and images (standards: HL7 and ASTM), and (c) application-level support - disease registries and implementation manuals (standards: HIPAA, HL7, ASTM, and ISO)	Yes, no, can’t say (if your answer is yes, +; no, -1; and can’t say, 0)
Section C: People-centric approach	Q4: Does the study mention stakeholder analysis/community needs assessment/with end users for the development of the initiative?	Consider: (a) Does the study cite previous studies published on gap analysis, stakeholder analysis, and qualitative surveys to understand the needs of the end users and (b) does the study involve a section or subsection wherein end users were involved in the development of a digital health initiative?	Yes, no, can’t say (if your answer is yes, +; no, -1; and can’t say, 0)
	Q5: Does the study mention the scope of collecting feedback from the end users?	Consider: (a) Was there any mention of the collection of feedback from the end users in the paper, and (b) in the case of applications, has feedback been collected and suitable changes been made to improve user-friendliness?	Yes, no, can’t say (if your answer is yes, +; no, -1; and can’t say, 0)

## Review

Results

Descriptive Analysis of the Number of Research Articles Published on Digital Health

**Figure 2:**
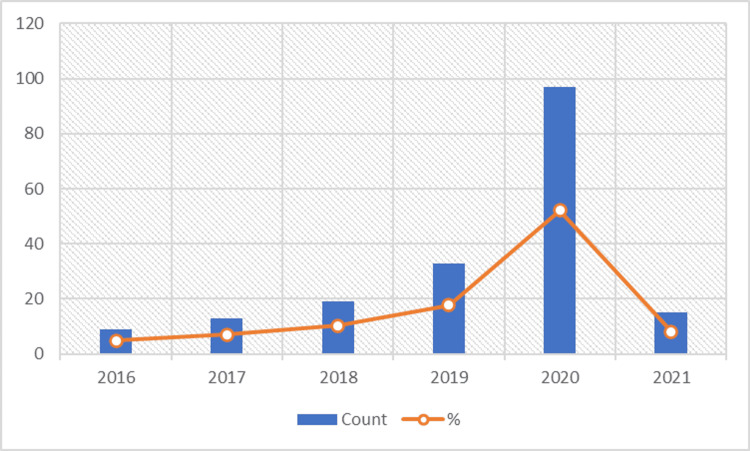
Year-wise trend in the number of publications Note: For the year 2021, data was collected only for the first six months as the data collection phase ended mid of 2021.

Our search strategy yielded a total of 563 articles on Medline. With a primary screening of article titles and abstracts, 186 articles were found to satisfy the eligibility criteria. From 2016 to 2020, the number of research articles published on digital health has been steadily increasing, and the trend over the past five years is visualized in Figure 2.

**Figure 3:**
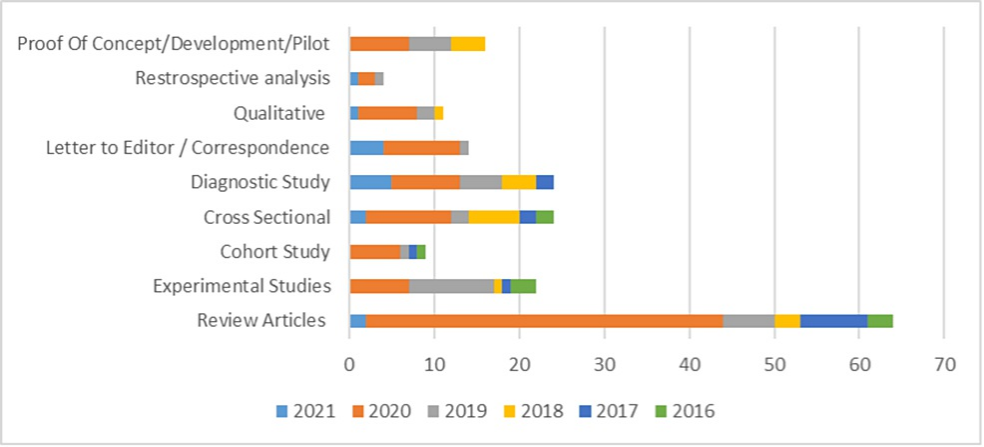
Design-wise and year-wise study stratification

When the studies were stratified based on study design, review articles and guidelines made up 65% of all the studies published on digital health in the past five years, with a maximum number of review articles published in 2020. Figure 3 visualizes the study design and year trend. The year-on-year trend presented an average of 10% increase in review articles, a 5% increase in letters to editors and correspondence, a 4% increase in diagnostic studies, and a 2%-3% increase in experimental studies, cohort studies, pilot studies, and development studies on digital health between 2016 and 2021.

**Figure 4:**
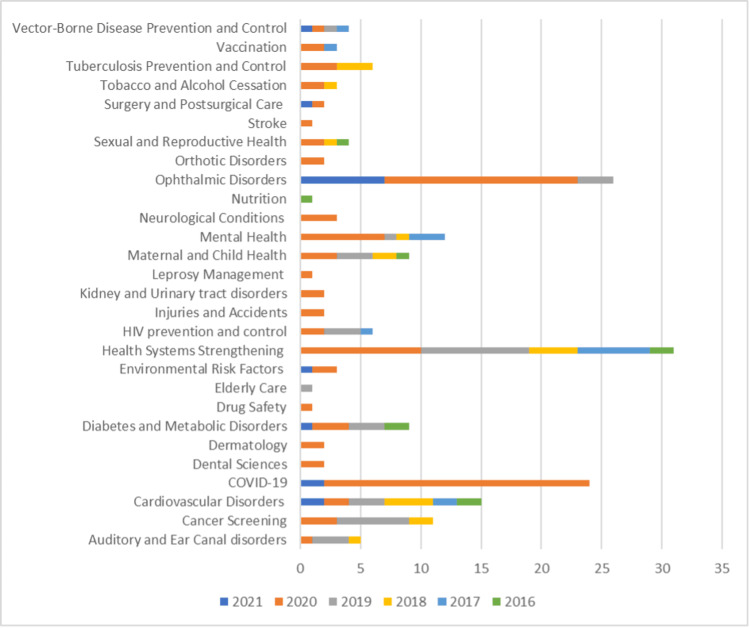
Stratification of studies on diseases and conditions or applications COVID-19: coronavirus disease 2019, HIV: human immunodeficiency virus

The studies were stratified based on diseases and conditions or applications for which digital initiative was primarily targeted across the five-year timeline (Figure 4). Health systems strengthening, which included initiatives such as capacity building among healthcare staff, geospatial analysis for improving access to healthcare services, and the use of data science to improve the availability of essential medicines, was the area wherein the highest digital health initiatives were focused. Ophthalmic disorders and COVID-19 were the second and third conditions to be frequently researched. For the year-on-year trend analysis of conditions or applications wherein digital health initiatives were primarily targeted, ophthalmic disorders and health systems strengthening recorded a <20% increase and cancer screening, cardiovascular disorders, and mental health recorded a 4%-6% increase on average from 2016 to 2021.

A negative year-on-year trend was seen for dental sciences, dermatology, drug safety, elderly care, HIV prevention and control, injuries and accidents, kidney and urinary tract disorders, leprosy management, neurological conditions, nutrition, orthotic disorders, sexual and reproductive health, stroke, tobacco and alcohol cessation, tuberculosis prevention and control, vaccination, and vector-borne disease prevention and control.

The study used the FITT framework to classify digital tools on tasks. The broad classification was health information exchange, data science, and surveillance. Further classification and definition with examples of each digital tool are presented in Table 2.

**Table 2:** Types of digital tools HIE: health information exchange, SMS: short message service

Task-wise classification	Subclassification	Definition	Example
HIE	Data sharing	Any tool a platform has to support data sharing between heterogeneous computer systems of different organizations [15]	Health information and management system
	Information delivery	Tool used for the delivery of information (preventive, curative) to the end users	SMSs sent for reminders of upcoming health visits
	Remote consultation	Consultation by remote telecommunications, generally for the purpose of the diagnosis or treatment of a patient at a site remote from the patient or primary physician [16]	Consultation using Skype and/or Zoom
	Intelligent diagnosis	An intelligent diagnosis system is one that is capable of identifying the nature of a problem by examining the observed symptoms and possibly an explanation or justify the same [17]	Algorithm-based diagnosis of risk of developing diabetes
Data science	Patient-generated health data	Health-related data created, gathered, or inferred by or from patients and for which the patient controls data collection and data sharing [18]	Digital diary
	Predictive analytics	Predictive analytics is a branch of advanced analytics that makes predictions about future outcomes using historical data combined with statistical modeling, data mining techniques, and machine learning [19]	Predictions on chances of raining
	Clinical decision support system	Tools designed to be a direct aid to clinical decision-making, in which the characteristics of an individual patient are matched to a computerized clinical knowledge base and patient-specific assessments or recommendations are then presented to the clinician for a decision [20]	Image-based computer-assisted screening of oral lesions for cancer screening
	Big data mining	Big data analytics covers the integration of heterogeneous data, data quality control, analysis, modeling, interpretation, and validation [21]	Using air pollution data to develop Air Quality Index
Surveillance	Risk screening	Continuous risk assessment of a condition or population through multiple screening surveys [22]	NA
	Real-time data collection and visualization	Ongoing collection of data and presentation of data in a pictorial or graphical format [23]	COVID-19 dashboards
	Contact tracing	Digitalization of identification and follow-up of persons who may have come into contact with a person infected with the infectious diseases [24]	NA

**Figure 5:**
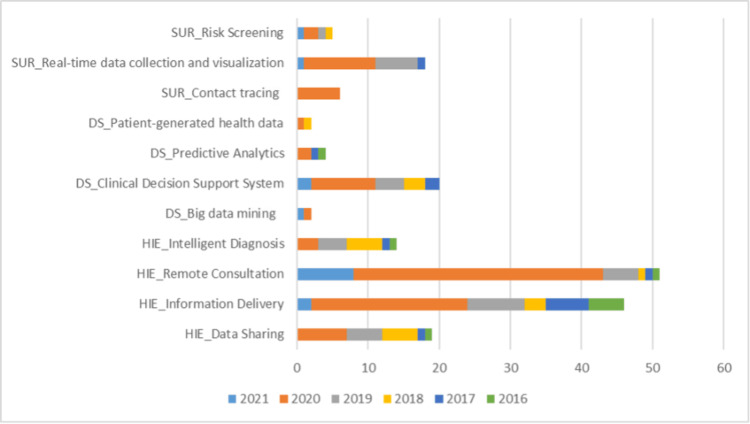
Stratification of studies based on digital tool used DS: data science, HIE: health information exchange, SUR: surveillance

The studies on remote consultation were most commonly reported, followed by information delivery systems and clinical decision support systems, as visualized in Figure 5.

**Figure 6:**
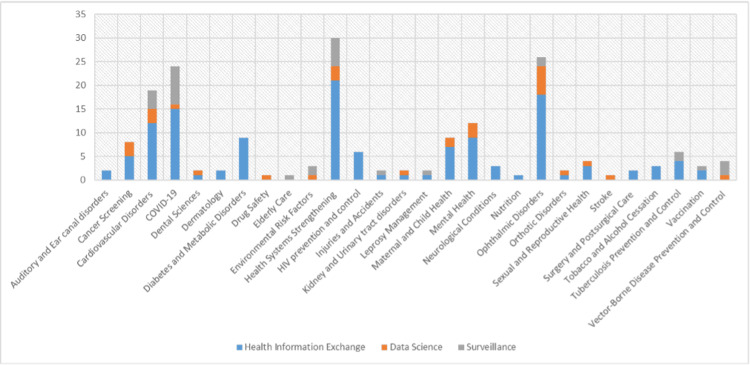
Cross tabulation of conditions and the type of digital tool used COVID-19: coronavirus disease 2019, HIV: human immunodeficiency virus

Cross tabulation of conditions and type of digital tool used is visualized in Figure 6. Health information exchange has been the most common digital tool for the majority of conditions, including all digital initiatives in metabolic disorders (diabetes), tobacco and alcohol cessation, and HIV prevention and control. Surveillance initiatives were commonly employed for vector-borne disease control, cardiovascular disorders, COVID-19, and health systems strengthening. Data science initiatives were common for ophthalmic disorders and health systems strengthening.

The year-on-year trend analysis of the digital tools used presented an average annual increase of 5% in health information exchange, with remote consultation recording a 9% average annual increase, followed by a 7% increase in health information delivery. Data science tools recorded a 0.5% annual increase, and under data science tools, only clinical decision support systems recorded a 2.5% average annual increase, whereas big data mining, predictive analysis, and patient-generated health data tools recorded a negative year-on-year trend. The surveillance tools usage trend presented a 0.5% average annual increase for 2016-2021, with real-time data collection and visualization tools showing a 2% increase.

The descriptive analysis highlights the following: (a) the COVID-19 pandemic has positively impacted digital health research with a 40% increase in the number of studies reported in 2020 when compared to 2019, (b) the three most common diseases and conditions wherein substantive digital health research has been focused are health systems strengthening, ophthalmic disorders, and COVID-19, and (c) remote consultation, health information delivery, and clinical decision support systems are the top three commonly developed tools from 2016 to 2021.

Assessment of Sustainability

Validation of sustainability assessment tool: We use inter-rater operability as an indicator to measure the ease of replicability of the tool. An independent rater assessed 30 randomly selected studies using a computer-generated list of the 87 articles assessed in section B of this study. Cohen’s unweighted Kappa was used to determine the inter-rater operability. The overall Kappa value was 0.806 (±0.088). The highest inter-rater operability was seen for Q1 (Does the study involve a multisectoral team?), with a Kappa value of 1 (100%), and the lowest inter-rater operability was seen for Q3 (Does the study mention adherence to any standards of data components, data interchange, and application-level support?), with a Kappa value of 0.54 (±0.036).

The Kappa values for the other questions were as follows: (a) Q2 (Does the study involve engagement with sectors other than health?), 0.91 (±0.066); (b) Q4 (Does the study mention stakeholder analysis/community needs assessment/with end users for the development of initiatives?), 0.84 (±0.042); and (C) Q5 (Does the study mention the scope of collecting feedback from the end users?), 0.71 (±0.06).

The interpretation of results was divided into studies published before the COVID-19 pandemic (2016-2019) and during the COVID-19 pandemic (2020-2021). Analysis was reported for the study design.

Experimental Study Design

All experimental studies before and during the pandemic had an active involvement of the IT and data management teams while developing the intervention and were either part of the team writing the manuscript or fully acknowledged, indicating that the multisectoral team was involved in developing and testing the initiatives.

Ten of 18 experimental studies involved the engagement of multiple sectors; for example, Swendeman et al. [25] included behavioral scientists, HIV care providers, and frontline health workers in the implementation of the study.

All the experimental studies mentioned adherence to data standards such as WHO or International Classification of Diseases (ICD) standards; however, adherence to data interchange standards such as HL7 was not mentioned.

All the experimental studies have either conducted gap analysis or referred to previously published authors’ papers on gap analysis and community needs assessment for the development of initiatives. All the experimental studies have mentioned feedback collection from end users and delivery providers and have mentioned changes made in digital initiatives upon receiving feedback.

Overall, the average sustainability of experimental studies on digital health was 80%, and there was no statistically significant difference in overall sustainability score between the studies published pre-pandemic (85.6%) and during the pandemic (76.4%) (p=0.33).

**Figure 7:**
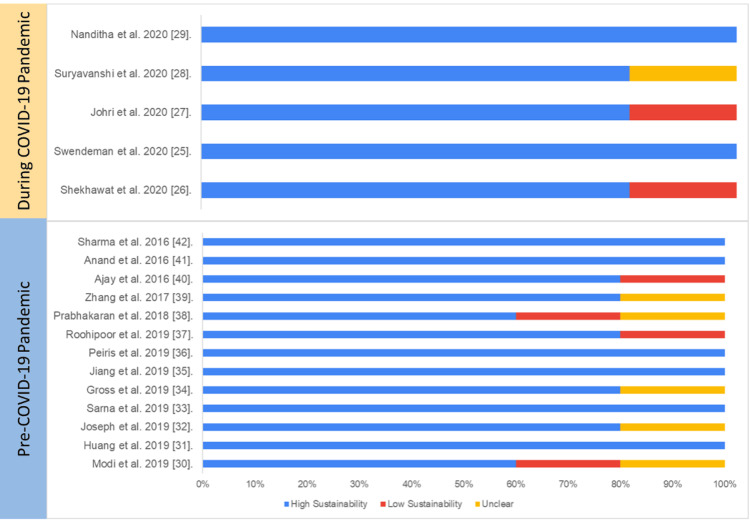
Assessment of sustainability (experimental studies) Blue: high sustainability, red: low sustainability, yellow: unclear sustainability COVID-19:coronavirus disease 2019

Table 3 and Figure 7 present the study-wise assessment score summary and percentage of sustainability scores based on the authors’ judgment.

**Table 3:** Assessment of sustainability (experimental studies) COVID-19: coronavirus disease 2019

	Author	Does the study involve a multisectoral team?	Does the study involve engagement with sectors other than health?	Does the study mention adherence to any standards of data components, data interchange, and application-level support?	Does the study mention stakeholder analysis/community needs assessment/with end users for the development of initiatives?	Does the study mention the scope of collecting feedback from the end users?
During COVID-19 pandemic	Shekhawat et al. (2020) [26]	Yes	No	Yes	Yes	Yes
	Swendeman et al. (2020) [25]	Yes	Yes	Yes	Yes	Yes
	Johri et al. (2020) [27]	Yes	No	Yes	Yes	Yes
	Suryavanshi et al. (2020) [28]	Yes	Can’t say	Yes	Yes	Yes
	Nanditha et al. (2020) [29]	Yes	Yes	Yes	Yes	Yes
Pre-COVID-19 pandemic	Modi et al. (2019) [30]	Yes	No	Yes	Yes	Can’t say
	Huang et al. (2019) [31]	Yes	Yes	Yes	Yes	Yes
	Joseph et al. (2019) [32]	Yes	Yes	Can’t say	Yes	Yes
	Sarna et al. (2019) [33]	Yes	Yes	Yes	Yes	Yes
	Gross et al. (2019) [34]	Yes	Can’t say	Yes	Yes	Yes
	Jiang et al. (2019) [35]	Yes	Yes	Yes	Yes	Yes
	Peiris et al. (2019) [36]	Yes	Yes	Yes	Yes	Yes
	Roohipoor et al. (2019) [37]	Yes	No	Yes	Yes	Yes
	Prabhakaran et al. (2018) [38]	Yes	No	Can’t say	Yes	Yes
	Zhang et al. (2017) [39]	Yes	Yes	Can’t say	Yes	Yes
	Ajay et al. (2016) [40]	Yes	No	Yes	Yes	Yes
	Anand et al. (2016) [41]	Yes	Yes	Yes	Yes	Yes
	Sharma et al. (2016) [42]	Yes	Yes	Yes	Yes	Yes

Cohort Study Design

Of the cohort studies, 80% had active involvement of the IT and data management teams while developing the intervention and were either part of the team writing the manuscript or fully acknowledged, indicating that the multisectoral team was involved in developing and testing the initiatives. The pattern of engagement with multisectoral was similar in studies reported before the COVID-19 pandemic and during the COVID-19 pandemic. However, 80% of the studies assessed did not show engagement of multiple sectors.

Of the cohort studies, 65% reported adherence to WHO/International Classification of Diseases (ICD) standards for the classification of diseases; however, studies have not mentioned adherence to data interchange standards such as HL7.

All the cohort studies assessed have mentioned gap analysis and needs assessment for the development of initiatives. About 60% mentioned feedback collection from end users and delivery providers and mentioned changes made in digital initiatives upon receiving feedback.

Overall, the average sustainability of cohort studies on digital health was 40%, and there was no statistically significant difference in overall sustainability score (if the score has been determined and the tool's validity has been assessed, this information is reported in a yes or no format) between the studies published pre-pandemic (25.3%) and during the pandemic (35.6%) (p=0.45).

**Figure 8:**
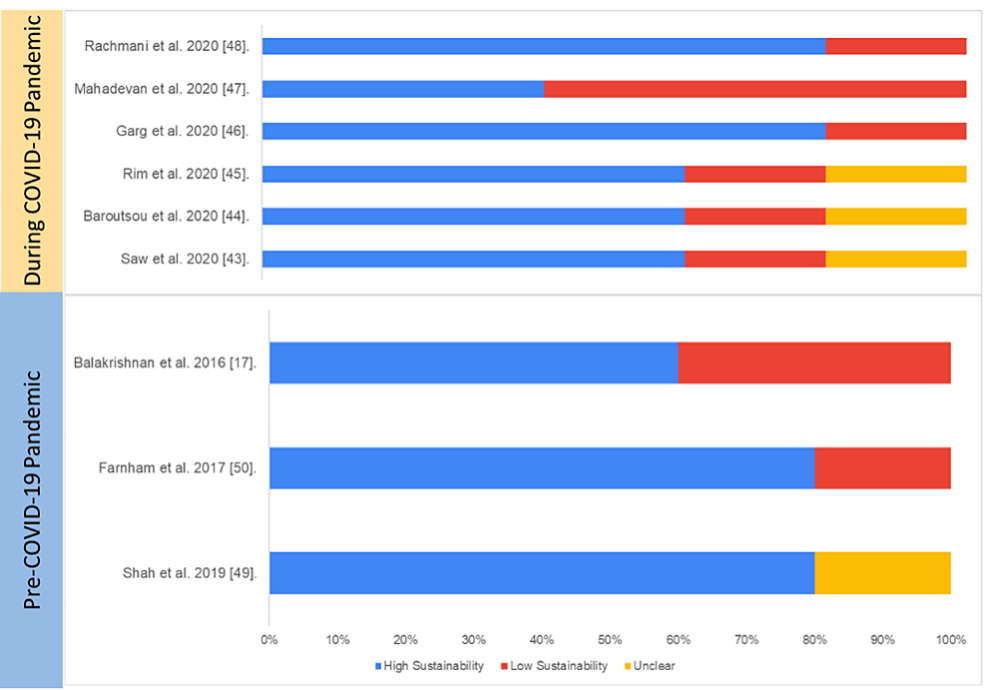
Assessment of sustainability (cohort studies) Blue: high sustainability, red: low sustainability, yellow: unclear sustainability COVID-19: coronavirus disease 2019

Table 4 and Figure 8 present the study-wise assessment score summary and percentage of sustainability scores based on the authors’ judgment.

**Table 4:** Assessment of sustainability (cohort studies) COVID-19: coronavirus disease 2019

	Author	Does the study involve a multisectoral team?	Does the study involve engagement with sectors other than health?	Does the study mention adherence to any standards of data components, data interchange, and application-level support?	Does the study mention stakeholder analysis/community needs assessment/with end users for the development of initiatives?	Does the study mention the scope of collecting feedback from the end users?
During COVID-19 pandemic	Saw et al. (2020) [43]	Yes	Can’t say	No	Yes	Yes
	Baroutsou et al. (2020) [44]	Yes	No	Can’t say	Yes	Yes
	Rim et al. (2020) [45]	Yes	No	Yes	Yes	Can’t say
	Garg et al. (2020) [46]	Yes	No	Yes	Yes	Yes
	Mahadevan et al. (2020) [47]	No	No	Yes	Yes	No
	Rachmani et al. (2020) [48]	Yes	No	Yes	Yes	Yes
Pre-COVID-19 pandemic	Shah et al. (2019) [49]	Yes	Yes	Can’t say	Yes	Yes
	Farnham et al. (2017) [50]	Yes	No	Yes	Yes	Yes
	Balakrishnan et al. (2016) [17]	No	No	Yes	Yes	Yes

Cross-Sectional Study Design

Of the cross-sectional studies, 25% had active involvement of the IT and data management teams while developing the intervention and were either part of the team writing the manuscript or fully acknowledged, indicating that the multisectoral team was involved in developing and testing the initiatives. The percentage of active involvement of the multisectoral team was higher in cross-sectional studies reported during COVID-19 as compared to studies reported pre-COVID-19. However, in 80% of cross-sectional studies assessed, engagement of multiple sectors was not seen.

Of the cross-sectional studies reported during the COVID-19 pandemic, 90% reported adherence to WHO/ICD standards for the classification of diseases, whereas only 10% of cross-sectional studies reported pre-COVID-19 reported adherence to WHO/ICD standards for the classification of diseases.

Of the cross-sectional studies, 30% have mentioned gap analysis and needs assessment for the development of initiatives. About 40% of the studies have mentioned feedback collection from end users and delivery providers and have mentioned changes made in digital initiatives upon receiving feedback.

Overall, the average sustainability of cross-sectional studies on digital health was 40%, and there was a statistically significant difference in overall sustainability score between the studies published pre-pandemic (45.3%) and during the pandemic (27.7%) (p=0.002).

**Figure 9:**
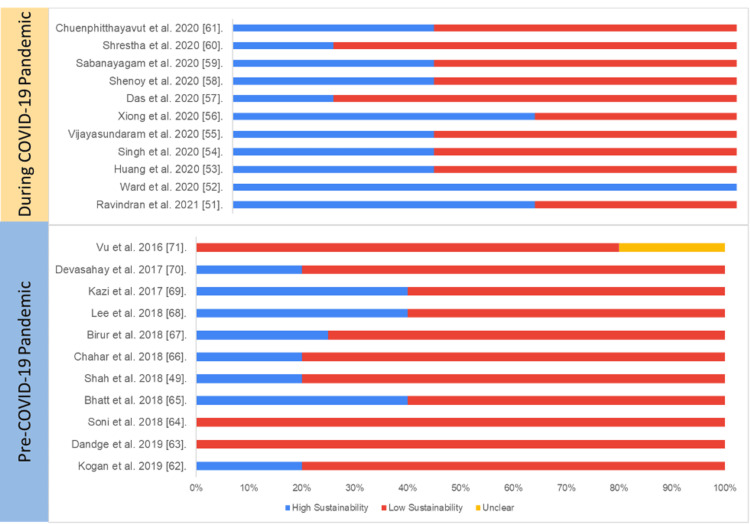
Assessment of sustainability (cross-sectional studies) Blue: high sustainability, red: low sustainability, yellow: unclear sustainability COVID-19: coronavirus disease 2019

Table 5 and Figure 9 present the study-wise assessment score summary and percentage of sustainability scores based on the authors’ judgment.

**Table 5:** Assessment of sustainability (cross-sectional studies) COVID-19: coronavirus disease 2019

	Author	Does the study involve a multisectoral team?	Does the study involve engagement with sectors other than health?	Does the study mention adherence to any standards of data components, data interchange, and application-level support?	Does the study mention stakeholder analysis/community needs assessment/with end users for the development of initiatives?	Does the study mention the scope of collecting feedback from the end users?
During COVID-19 pandemic	Ravindran et al. (2021) [51]	No	No	Yes	Yes	Yes
	Ward et al. (2020) [52]	Yes	Yes	Yes	Yes	Yes
	Huang et al. (2020) [53]	No	No	Yes	No	Yes
	Singh et al. (2020) [54]	No	No	Yes	No	Yes
	Vijayasundaram et al. (2020) [55]	No	No	Yes	No	Yes
	Xiong et al. (2020) [56]	Yes	Yes	Yes	No	No
	Das et al. (2020) [57]	No	No	Yes	No	No
	Shenoy et al. (2020) [58]	No	No	No	Yes	Yes
	Sabanayagam et al. (2020) [59]	Yes	No	Yes	No	No
	Shrestha et al. (2020) [60]	No	No	Yes	No	No
	Chuenphitthayavut et al. (2020) [61]	Yes	No	Yes	No	No
Pre-COVID-19 pandemic	Kogan et al. (2019) [62]	Yes	No	No	No	No
	Dandge et al. (2019) [63]	No	No	No	No	No
	Soni et al. (2018) [64]	No	N0	No	No	No
	Bhatt et al. (2018) [65]	No	No	No	Yes	Yes
	Shah et al. (2018) [49]	Yes	No	No	No	No
	Chahar et al. (2018) [66]	No	No	Yes	No	No
	Birur et al. (2018) [67]	No	No	No	Can’t say	Yes
	Lee et al. (2018) [68]	Yes	No	No	No	Yes
	Kazi et al. (2017) [69]	Yes	No	No	Yes	No
	Devasahay et al. (2017) [70]	Yes	No	No	No	No
	Vu et al. (2016) [71]	Can’t say	No	No	No	No

Diagnostic Studies

Of the diagnostic studies, 70% had active involvement of the IT and data management teams while developing the intervention and were either part of a team writing the manuscript or fully acknowledged, indicating that the multisectoral team was involved in developing and testing the initiatives. The percentage of active involvement of multisectoral teams was higher in diagnostic studies reported during the COVID-19 pandemic compared to studies reported pre-COVID-19 pandemic. However, in 95% of diagnostic studies assessed, engagement of multiple sectors was not seen.

Of the diagnostic studies, 60% reported adherence to WHO/ICD standards for the classification of diseases; the majority of studies reported during the COVID-19 pandemic were able to provide information on adherence to standards.

Of the diagnostic studies assessed, 25% have mentioned gap analysis and needs assessment for the development of initiatives. About 30% of the studies have mentioned feedback collection from end users and delivery providers and have mentioned changes made in digital initiatives upon receiving feedback.

Overall, the average sustainability of diagnostic studies on digital health was 45%, and there was no statistically significant difference in overall sustainability score between the studies published pre-pandemic (45%) and during the pandemic (55%) (p=0.5)

**Figure 10:**
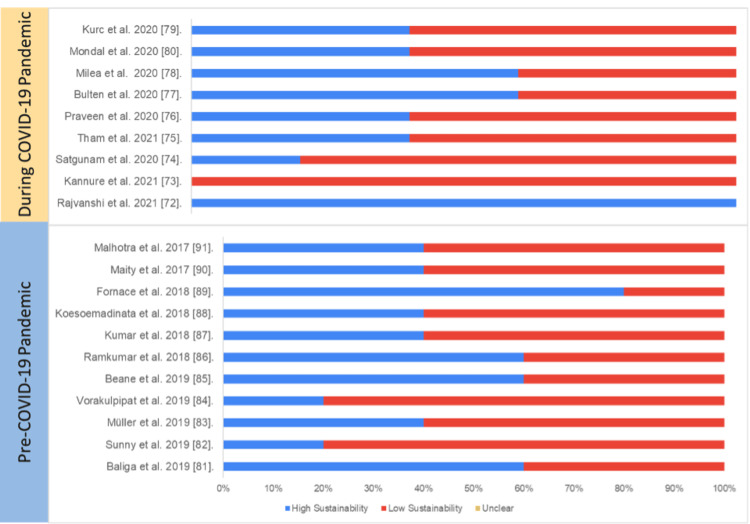
Assessment of sustainability (diagnostic studies) Blue: high sustainability, red: low sustainability, yellow: unclear sustainability COVID-19: coronavirus disease 2019

Table 6 and Figure 10 present the study-wise assessment score summary and percentage of sustainability scores based on the authors’ judgment.

**Table 6:** Assessment of sustainability (diagnostic studies) COVID-19: coronavirus disease 2019

	Author	Does the study involve a multisectoral team?	Does the study involve engagement with sectors other than health?	Does the study mention adherence to any standards of data components, data interchange, and application-level support?	Does the study mention stakeholder analysis/community needs assessment/with end users for the development of initiatives?	Does the study mention the scope of collecting feedback from the end users?
During COVID-19 pandemic	Rajvanshi et al. (2021) [72]	Yes	Yes	Yes	Can’t say	Yes
	Kannure et al. (2021) [73]	No	No	No	No	No
	Satgunam et al. (2020) [74]	No	No	Yes	No	No
	Tham et al. (2021) [75]	Yes	No	Yes	No	No
	Praveen et al. (2020) [76]	Yes	No	Yes	No	No
	Bulten et al. (2020) [77]	No	No	Yes	Yes	Yes
	Milea et al. (2020) [78]	No	No	Yes	Yes	Yes
	Kurc et al. (2020) [79]	Yes	No	Yes	No	No
	Mondal et al. (2020) [80]	No	No	Yes	No	Yes
Pre-COVID-19 pandemic	Baliga et al. (2019) [81]	Yes	No	No	Yes	Yes
	Sunny et al. (2019) [82]	Yes	No	No	No	No
	Müller et al. (2019) [83]	Yes	No	Yes	No	No
	Vorakulpipat et al. (2019) [84]	Yes	No	No	No	No
	Beane et al. (2019) [85]	Yes	No	No	Yes	Yes
	Ramkumar et al. (2018) [86]	Yes	No	No	Yes	Yes
	Kumar et al. (2018) [87]	Yes	No	Yes	No	No
	Koesoemadinata et al. (2018) [88]	Yes	No	Yes	No	No
	Fornace et al. (2018) [89]	Yes	No	Yes	Yes	Yes
	Maity et al. (2017) [90]	Yes	No	Yes	No	No
	Malhotra et al. (2017) [91]	No	No	No	Yes	Yes

Pilot Studies and Development Studies

We used the definition given by Stewart [92] for pilot and development studies as a “small study to test research protocols, data collection instruments, sample recruitment strategies, and other research techniques in preparation for a larger study.” However, pilot randomized studies were included in experimental studies.

Of the pilot and development studies, 75% had active involvement of the IT and data management teams while developing the intervention and were either part of the team writing the manuscript or fully acknowledged, indicating that the multisectoral team was involved in developing and testing the initiatives. The percentage of active involvement of multisectoral teams was similar for studies reported during the COVID-19 pandemic and studies reported before the COVID-19 pandemic. However, in 95% of diagnostic studies assessed, engagement of multiple sectors was not seen.

Of the pilot and development studies, 80% reported adherence to WHO/ICD standards for the classification of diseases.

Of the pilot and development studies assessed, 50% have mentioned gap analysis and needs assessment for the development of initiatives. Similarly, 50% of the studies have mentioned feedback collection from end users and delivery providers and have mentioned changes made in digital initiatives upon receiving feedback.

Overall, the average sustainability of pilot and development studies on digital health was 65%, and there was no statistically significant difference in overall sustainability score between the studies published pre-pandemic (56%) and during the pandemic (63%) (p=0.28).

**Figure 11:**
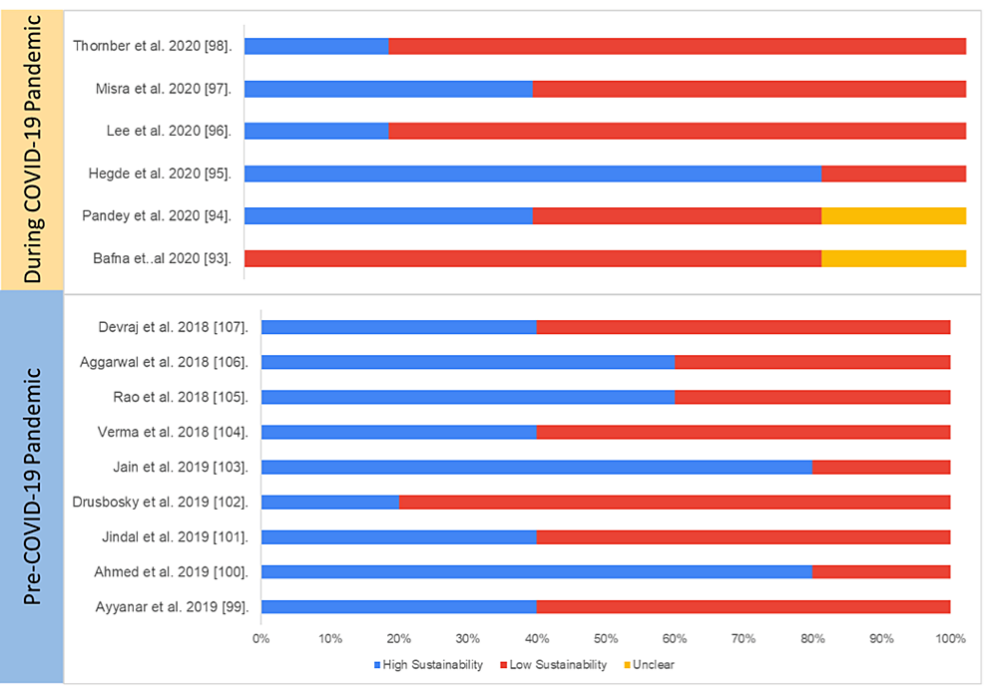
Assessment of sustainability (pilot and development studies) Blue: high sustainability, red: low sustainability, yellow: unclear sustainability COVID-19: coronavirus disease 2019

Table 7 and Figure 11 present the study-wise assessment score summary and percentage of sustainability scores based on the authors’ judgment.

**Table 7:** Assessment of sustainability (pilot and development studies) COVID-19: coronavirus disease 2019

	Author	Does the study involve a multisectoral team?	Does the study involve engagement with sectors other than health?	Does the study mention adherence to any standards of data components, data interchange, and application-level support?	Does the study mention stakeholder analysis/community needs assessment/with end users for the development of initiatives?	Does the study mention the scope of collecting feedback from the end users?
During COVID-19 pandemic	Bafna et al. (2020) [93]	No	No	Yes	No	No
	Pandey et al. (2020) [94]	No	No	No	Yes	Yes
	Hegde et al. (2020) [95]	Yes	No	No	No	No
	Lee et al. (2020) [96]	Yes	No	Yes	No	No
	Misra et al. (2020) [97]	Yes	No	Yes	Yes	Yes
	Thornber et al. (2020) [98]	Yes	No	No	Yes	Yes
Pre-COVID-19 pandemic	Ayyanar et al. (2019) [99]	Yes	No	Yes	No	No
	Ahmed et al. (2019) [100]	No	No	Yes	No	No
	Jindal et al. (2019) [101]	Yes	No	Yes	Yes	Yes
	Drusbosky et al. (2019) [102]	Yes	No	Yes	No	No
	Jain et al. (2019) [103]	Yes	No	No	Yes	Yes
	Verma et al. (2018) [104]	No	Yes	Yes	No	No
	Rao et al. (2018) [105]	Yes	No	Yes	Yes	Yes
	Aggarwal et al. (2018) [106]	Yes	No	Yes	No	No
	Jindal et al. (2018) [107]	Yes	No	Yes	Yes	Yes

Retrospective Analysis Studies

**Figure 12:**
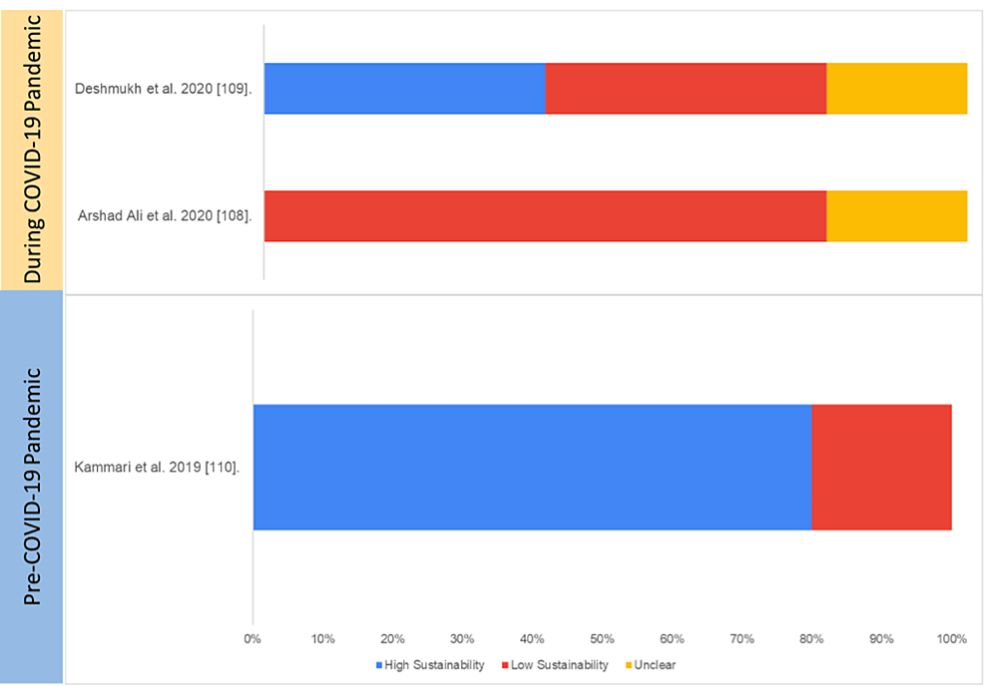
Assessment of sustainability (retrospective analysis studies) Blue: high sustainability, red: low sustainability, yellow: unclear sustainability COVID-19: coronavirus disease 2019

Three studies were assessed for sustainability using retrospective analysis study design as shown in Table 8 and Figure 12. Kammari et al. conducted a study pre-COVID-19 pandemic that demonstrated 80% chances of sustainability.

**Table 8:** Assessment of sustainability (retrospective analysis studies) COVID-19: coronavirus disease 2019

	Author	Does the study involve a multisectoral team?	Does the study involve engagement with sectors other than health?	Does the study mention adherence to any standards of data components, data interchange, and application-level support?	Does the study mention stakeholder analysis/community needs assessment/with end users for the development of initiatives?	Does the study mention the scope of collecting feedback from the end users?
During COVID-19 pandemic	Arshad Ali et al. (2020) [108]	No	No	Can’t say	No	No
	Deshmukh et al. (2020) [109]	Can’t say	No	No	Yes	Yes
Pre-COVID-19 pandemic	Kammari et al. (2019) [110]	Yes	No	Yes	Yes	Yes

The assessment of sustainability analysis indicates the following: (a) experimental studies and cohort studies had incorporated factors contributing to the sustainability and involvement of teams and sectors, and feedback was reported across the majority of studies irrespective of whether it was conducted pre-COVID-19 pandemic or during the COVID-19 pandemic; (b) cross-sectional studies conducted during the pandemic improved on parameters of assessment of sustainability, with a statistically significant difference between sustainability assessment during the COVID-19 pandemic and before the COVID-19 pandemic; and (c) diagnostic studies and pilot and development studies incorporated limited factors contributing to sustainability irrespective of whether it was conducted pre-COVID-19 pandemic or during the COVID-19 pandemic.

Discussion

To our knowledge, this study is the first to systematically analyze and present evidence from the sustainability perspective of digital health research initiatives across South Asia. A systematic review by Bassi et al. [111] in 2018 looked at the current status and future perspectives of mHealth from health systems perspectives; the review highlighted the poor quality of evidence generated through mHealth research. Another review by Bassi et al. [112] in 2020 presented the review of COVID-19-related mobile applications and highlighted gaps to inform the development of future mHealth initiatives, wherein the functionality of mobile applications was assessed to adjunct risk assessment efforts. Another systematic review by Kondylakis et al. [113] raised concerns about the quality of research studies published on the development and implementation of COVID-19 mobile applications.

The COVID-19 pandemic saw a huge increase in research on digital health initiatives; our study hypothesis was based on the increased number that has affected the sustainability of the research initiatives and the intrinsic factors of research initiatives such as multisectoral involvement, gap analysis, and stakeholder engagement.

In this paper, we descriptively analyzed the impact of the COVID-19 pandemic on the volume of research on digital health. To ascertain sustainability, we searched for standardized tools available for sustainability assessment; however, the tools did not suit the needs presented in this study. This led to the development of a simple tool for the assessment of sustainability. The assessment of sustainability was undertaken for 87 articles, and the tool can determine intrinsic sustainability factors and give summary estimates on how well the authors incorporate sustainability in digital health research.

In our study, we found that the number of digital health intervention research increased significantly during the COVID-19 pandemic, and most authors took the initiative to have oversight on the sustainability of their digital health initiatives. Contrary to our hypothesis, the sustainability score of cross-sectional studies was higher for studies published during the COVID-19 pandemic as compared to studies published before the COVID-19 pandemic.

According to our study findings, it is clear that the choice of digital tool, disease, and study design vary highly based on study objectives and research; however, the patterns presented over five years show higher research interest in ophthalmic disorders and health systems strengthening, while health information exchange tools have been exclusively used. The underlying reasons can be further explored and taken up for further research and help derive recommendations at the policy level.

## Conclusions

We conclude that the COVID-19 pandemic had a silver lining, and it positively impacted digital health research by improving the number of research initiatives undertaken in South Asia and with researchers able to develop a long-term vision for digital health initiatives.

The momentum and interest in digital health gained due to the COVID-19 pandemic should be sustained in the post-pandemic world, and with our sustainability analysis, there is certain confidence that researchers are able to develop a vision to sustain the initiative. However, this depends on extrinsic factors such as the availability of skilled manpower, a conducive policy environment, and access to the internet and hardware among the target population.
